# Efficacy of Metarhizium anisopliae isolate MAX-2 from Shangri-la, China under desiccation stress

**DOI:** 10.1186/1471-2180-14-4

**Published:** 2014-01-03

**Authors:** Zi-Hong Chen, Ling Xu, Feng-lian Yang, Guang-Hai Ji, Jing Yang, Jian-Yun Wang

**Affiliations:** 1 Department of Resources and Environmental Sciences, Baoshan College, Baoshan, Yunnan 678000, China; 2 Institute of Insect Resources, College of Plant Science and Technology, Huazhong Agricultural University, Wuhan 430070, China; 3 Key Laboratory of Agro-biodiversity and Pest Management of Education Ministry of China, Yunnan Agricultural University, Kunming, Yunnan 650201, China

**Keywords:** Biological control, *Metarhizium anisopliae*, *Tenebrio molitor*, Desiccation stress, Moisture level

## Abstract

**Background:**

*Metarhizium anisopliae*, a soil-borne entomopathogen found worldwide, is an interesting fungus for biological control. However, its efficacy in the fields is significantly affected by environmental conditions, particularly moisture. To overcome the weakness of *Metarhizium* and determine its isolates with antistress capacity, the efficacies of four *M. anisopliae* isolates, which were collected from arid regions of Yunnan Province in China during the dry season, were determined at different moisture levels, and the efficacy of the isolate MAX-2 from Shangri-la under desiccation stress was evaluated at low moisture level.

**Results:**

*M. anisopliae* isolates MAX-2, MAC-6, MAL-1, and MAQ-28 showed gradient descent efficacies against sterile *Tenebrio molitor* larvae, and gradient descent capacities against desiccation with the decrease in moisture levels. The efficacy of MAX-2 showed no significant differences at 35% moisture level than those of the other isolates. However, significant differences were found at 8% to 30% moisture levels. The efficacies of all isolates decreased with the decrease in moisture levels. MAX-2 was relatively less affected by desiccation stress. Its efficacy was almost unaffected by the decrease at moisture levels > 25%, but slowly decreased at moisture levels < 25%. By contrast, the efficacies of other isolates rapidly decreased with the decrease in moisture levels. MAX-2 caused different infection characteristics on *T. molitor* larvae under desiccation stress and in wet microhabitat. Local black patches were found on the cuticles of the insects, and the cadavers dried without fungal growth under desiccation stress. However, dark black internodes and fungal growth were found after death of the insects in the wet microhabitat.

**Conclusions:**

MAX-2 showed significantly higher efficacy and superior antistress capacity than the other isolates under desiccation stress. The infection of sterile *T. molitor* larvae at low moisture level constituted a valid laboratory bioassay system in evaluating *M. anisopliae* efficacy under desiccation stress.

## Background

Environmental concern and health risks associated with chemical insecticides have stimulated efforts to explore the use of fungi for biological control [1]. *Metarhizium anisopliae* (Metschnikoff) Sorokin is a fungus that is often found in soil, and can infect more than 200 species of insects [2]. This fungus is one of the first fungi used in biological control experiments.

However, *M. anisopliae* is less virulent in the field than in the laboratory [3,4] because the environmental conditions in the soil may diminish its pathogenicity. Fungi are highly dependent on the ambient microclimate. The performance of *M. anisopliae* products is affected by various environmental factors, such as soil moisture, air and soil temperatures, air relative humidity, and solar UV radiation.

The conidia of *M. anisopliae* attach to the cuticle of the host via germ tubes*.* The conidia germinate and directly penetrate the hyphae into the body integuments, and grow into the haemocoel, where they produce a blend of organic compounds that cause internal mechanical damage, nutrient depletion, and death. For successful infection, optimum moisture is needed for spores to germinate after attachment to the hosts. Germination, germ tube extension, and infection of *M. anisopliae* are optimized at Relative Humidity (RH) > 95% and temperatures between 20°C and 30°C [5]. Neutral trehalase has an important function in environmental stress response in many organisms, including *Metarhizium* spp. [6].

The successful development of entomopathogenic fungi as biological control agents significantly depends on the selection of highly efficient isolates, and the fungi must be adapted to the environmental conditions of the area where they are to be employed [7]. A successful microbial insecticide should possess desirable characteristics, such as high spore germination, high production, and high virulence [8]. The virulence of *M. anisopliae* against pests significantly varies among isolates [9]. The low virulence and low tolerance to adverse conditions in the field limit their applications [10]. More efforts should be made in obtaining *Metarhizium* isolates with high virulence and antistress capacity to overcome environmental stress. In our pre-experiment, *Metarhizium* isolates were obtained from arid regions of Yunnan Province in China during the dry season and identified (data not shown). One *M. anisopliae* isolate, MAX-2, which was obtained from Shangri-la (3200 m to 4100 m above sea level), showed high activities under desiccation stress.

This study aimed to evaluate the capacity of *M. anisopliae* isolate MAX-2 for infection under desiccation stress, and develop a valid laboratory bioassay system in testing the efficacy of *M. anisopliae* under desiccation stress with sterile *Tenebrio molitor* L. (yellow mealworm) larvae in a substrate with low moisture content. The efficacy of *M. anisopliae* isolate MAX-2 and its potential for controlling pests in desiccation environment were discussed.

## Results

### Sterile culture of host insects

**Figure 1:**
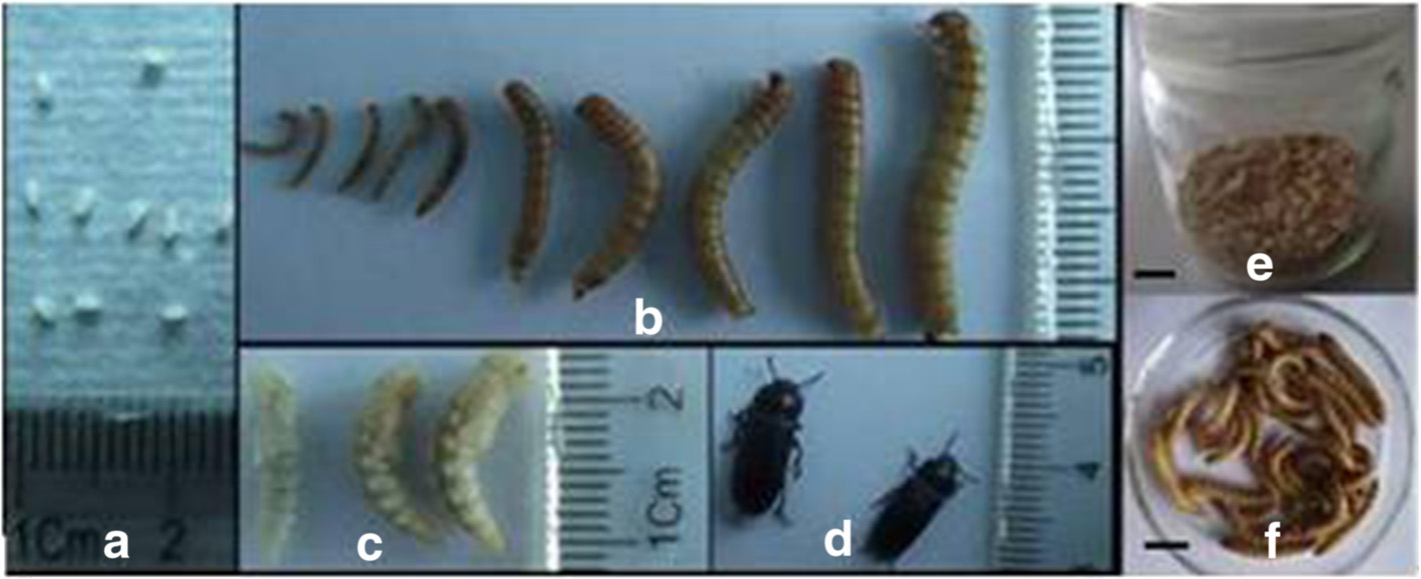
**Lifestyle of *T. molitor *and the larvae used for experiments.** Note: **a**-**d** showed the lifecycle of *T. molitor*, **a** for egg; **b** for larva; **c** for pupae; **d** for adult; **e** showed the larvae reared in the sterile wheat bran substrate; **f** showed the larvae used for experiments. Bar in **e** and **f** = 1 cm.

*T. molitor* larvae were successfully reared in sterile wheat bran substrates with 15% moisture content at 25°C under natural day light, and cultured for more than five generations before use for the tests (Figure 1e). The microbes on the larval surface were diluted from generation to generation, and the larvae were relatively sterile. The larvae used for tests were cultured on sterile wheat bran with 50% moisture content to investigate their sterility. *T. molitor* larvae were alive after 15 d, and no microbial growth was observed. This result suggests the absence of microbial contamination. Thus, the efficacy tests of *M. anisopliae* did not exhibit microbial interference.

*T. molitor* has a life cycle that consists of four stages, namely, egg (Figure 1a), larva (Figure 1b), pupa (Figure 1c), and adult (Figure 1d). They can complete their life cycle under desiccation stress, in which the larval stage exhibits relatively high desiccation endurance. The life cycle of *T. molitor* can encompass four months to several years, depending on the number and duration of the instars [11]. Under the experimental conditions, the larvae had 13 instars and pupated at 13th instar larvae. Figure 1b shows some larvae at various instars from third to 10th instar larvae.

### Conidial germination rate of M. anisopliae isolates at different moisture levels

**Figure 2:**
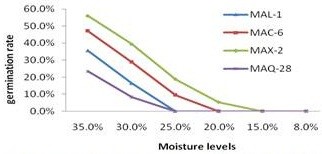
**Conidial germination rate of *M. anisopliae *** **isolates at different moisture levels.**

Conidial germination of all tested *M. anisopliae* isolates, namely, MAX-2, MAL-1, MAC-6, and MAQ-28, was positively influenced by moisture contents of the substrates (Figure 2). After 24 h of culture, no germination occurred in the substrates with low moisture contents (8% and 15%) for all the *M. anisopliae* applied treatments, and only MAX-2 had a poor germination rate of approximately 5% at 20% moisture level. The conidial germination rates of the isolates improved with the moisture levels, and reached 56% for MAX-2, 47.1% for MAC-6, 35.6% for MAL-1, and 23.4% for MAQ-28 at 35% moisture level.

### Efficacy of M. anisopliae isolates against T. molitor larvae at different moisture levels

All the tested *M. anisopliae* isolates inflicted mycoses on *T. molitor* larvae and caused 100% mortality when cultured in substrates with high moisture content (≥ 40%) at 25°C in our pre-experiment (data not shown). The efficacies of isolates were tested by separately inoculating their conidia (5?×?10^8^ conidia/g) in wheat bran substrates with 8% to 35% moisture contents, and sterile *T. molitor* larvae were cultured in the substrates with *M. anisopliae* conidia at 25°C. The four isolates had gradient descent efficacies, and MAX-2 showed relatively high efficacy at most of the tested moisture levels. Lower moisture levels significantly enhanced the difference and highlighted the superiority of the efficacy of MAX-2 under desiccation stress (Table 1).

**Table 1:** **Multiple range comparison of hosts’ mortality rates for *M. anisopliae *** **isolates at different moisture levels**

**Moisture levels**	**MAX-2 (%)**	**MAC-6 (%)**	**MAL-1 (%)**	**MAQ-28 (%)**	**F**	**df**	**P**
35%	100.00?±?0a	100.00?±?0a	100.00?±?0a	95.33?±?2.08a	15.08	3, 11	0.31
30%	100.00?±?0a	83.33?±?1.53b*	74.33?±?1.53b**	61.67?±?1.53b**	445.27	3, 11	0.00
25%	92.33?±?2.08a	56.67?±?1.53c**	47.67?±?3.21c**	29.00?±?1.00c**	470.74	3, 11	0.00
20%	78.00?±?2.65b	40.33?±?0.58d**	28.00?±?2.65d**	10.67?±?1.53d**	587.11	3, 11	0.00
15%	57.33?±?2.52c	19.00?±?1.00e**	8.00?±?2.00e**	0.00?±?0.00d**	682.62	3, 11	0.00
8%	41.33?±?1.53d	4.00?±?1.00f**	0.00?±?0.00e**	0.00?±?0.00d**	1452.80	3, 11	0.00
F1	530.070	3509.562	1148.687	2663.893	-	-	-
df1	5, 17	5, 17	5, 17	5, 17	-	-	-
P1	0.00	0.00	0.00	0.00	-	-	-

Data are expressed as means?±?Standard deviations (SD). Within each column, different letters indicate differences significant (P < 0.05) and the same letters indicate no statistic differences. Within each row, one *means the difference is significant (P < 0.05); two *means the difference is very significant (P < 0.01); no *means no statistic difference. The values of F, df, P are results of comparison among different isolates within each row (the same moisture level). And the values of F1, df1, P1 are results of comparison among different moisture levels within each column (the same isolate).

After 15 d of inoculation, the mortalities of *T. molitor* larvae reached 100% for all the isolates, except MAQ-28 (95% mortality) in the substrate with 35% moisture content. The efficacies between MAX-2 and other isolates showed no significant difference. However, the efficacies differed significantly between MAX-2 and other isolates at moisture levels of 8% to 30%. MAX-2 had the highest efficacy, whereas MAQ-28 had the lowest efficacy. MAX-2 maintained 100% mortality at 30% moisture level, whereas the efficacies of other isolates decreased. The mortalities for MAC-6, MAL-1, and MAQ-28 continued to decrease drastically with the decrease in moisture levels, and reached zero or close to zero at 8% moisture level. However, the mortality for MAX-2 slowly decreased with the decrease in moisture levels, and maintained medium mortality of 41% at 8% moisture level.

*T. molitor* larvae were healthy in control treatments with different moisture levels (8% to 35%) and continued their life cycle.

### Infection characteristics of MAX-2 under desiccation stress

The efficacies of all isolates decreased with the decrease in moisture levels, but the efficacy of MAX-2 was less affected by desiccation stress (Table 1). The efficacy of MAX-2 was almost unaffected by the decrease in moisture levels?>?25%, and no statistical difference was observed among higher moisture levels from 25% to 35%. Its efficacy slowly decreased with the decrease in moisture levels < 25%, and a significant difference was observed among lower moisture levels from 8% and 20%. The efficacy of MAC-6 significantly differed among all moisture levels from 8% to 35%. The efficacy of MAL-1 significantly differed among higher moisture levels (from 20% to 35%), but no significant difference was observed between lower moisture levels (8% and 15%). The efficacy of MAQ-28 significantly differed among higher moisture levels (from 25% to 35%), but no significant difference was observed among lower moisture levels (from 8% to 20%). These results suggest that the four isolates with gradient descent efficacies also had gradient descent capacities against desiccation, and MAX-2 had significantly higher antistress capacity under desiccation stress than the other isolates.

**Figure 3:**
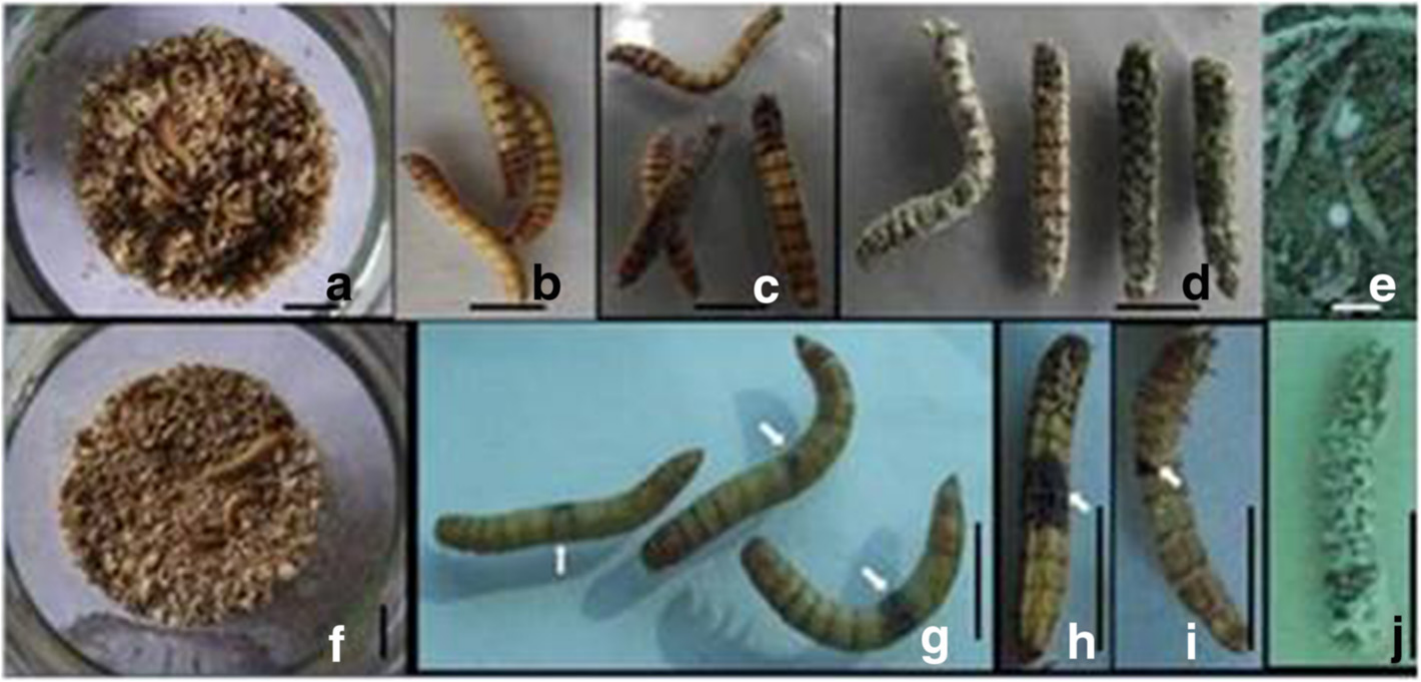
**The symptoms of T. molitor larvae infected by *M. anisopliae *isolate MAX-2.** Note: **a**-**e**, in the wet microhabitat; **f**-**j**, under desiccation stress. Bar = 1 cm **(a-j)**. The arrows in **g**, **h**, and **i** indicated the local black patches on the cuticles under desiccation stress.

MAX-2 caused similar symptoms to other isolates in the wet microhabitat (substrate with 35% moisture content; Figure 3a). *T. molitor* larvae exhibited bradykinesia, and the internodes of insects turned slightly brown in the early stage of infection (2 d to 3 d post-inoculation; Figure 3b). The internodes gradually became dark black, and the larvae died within the following 2 d (Figure 3c). White mycelia sprang up and gradually covered the cadavers approximately 10 d after inoculation. The conidia formed, and the larval surface turned green after another 1 d to 2 d (Figure 3d). The substrate also showed white mycelia, which gradually turned light green during the course of infection. This phenomenon suggests that new conidia formed and added to the initial inoculum concentration, thereby resulting in a large number of inocula around the larvae (Figure 3e).

In the dry microhabitat (substrate with 8% moisture content; Figure 3f), MAX-2 exhibited medium efficacy (41% mortality), whereas the other isolates showed no efficacies or very low efficacies (< 5% mortality). Similar to the observations in the wet microhabitat, *T. molitor* larvae exhibited bradykinesia in the dry microhabitat, but the larvae exhibited local black patches on the cuticles 3 d to 4 d after inoculation (Figure 3g). The local black patches gradually extended to one to two somites, and the larvae became slower, died, and dried (Figure 3h). However, no mycelium or conidia emerged on the insect cadavers and substrates when kept in dry substrate all the time. This result suggests that the moisture level was too low to facilitate mycelial or conidial growth. However, when the cadavers were transferred to a moist filter, mycelium and conidia rapidly emerged on their surface within 2 d to 3 d (Figure 3j). In addition, few larvae completed exuviation and survived even when local black patches appeared on the shell (Figure 3i).

## Discussion

### A valid laboratory bioassay system for evaluating M. anisopliae efficacy under desiccation stress

Water stress tolerance of fungal strains is usually evaluated using various salts to create different water potential scenarios. However, in testing the virulence of the strains, the salt can affect the life cycles of hosts. In this paper, a novel laboratory bioassay system was used to test the efficacy of *M. anisopliae* under desiccation stress on *T. molitor* larvae in dry substrate.

Extreme environmental trials of pathogens demand specific hosts. The yellow mealworm, *T. molitor*, is a freeze-susceptible, stored product pest. When provided with sufficient food supply, *T. molitor* larvae have low humidity tolerance and can survive under relatively xeric conditions because of their ability to metabolize water from ingested food [12].

Clopton et al. [13] sterilized adult and larval *T. molitor* by incubation at 36°C to 37°C for 5 d to eliminate the effect of existing gregarine infections on the tests. In the present study, the host insects were cultured and sterilized by generational dilution in sterile wheat bran substrates, and the insects were almost fully sterilized when given enough generation culture. This new method may provide host insects for strict experimental infections.

The efficacy of *M. anisopliae* under desiccation stress was tested in dry wheat bran substrate with initial moisture content of 8%. At this low moisture level, *M. anisopliae* was difficult to grow, but the isolate MAX-2 was still active, whereas the other isolates showed very low efficacy. This result suggests that the infection of sterile *T. molitor* larvae in wheat bran substrates with low moisture content could constitute a valid laboratory bioassay system to study *M. anisopliae* efficacy under desiccation stress.

### Efficacy of M. anisopliae isolate MAX-2

This study demonstrated that *M. anisopliae* isolate MAX-2 had pathogenicity against *T. molitor* larvae in all the tested moisture levels, particularly lower moisture levels, and showed relatively high tolerance to desiccation stress.

Daoust et al. [14] indicated that the efficacy of *M. anisopliae* against insects depends on conidial germination. Conidial germination of all tested isolates in the present study showed a tendency to decrease with the decrease in substrate moisture content within the tested scope (8% to 35%). The mortality of larvae for the isolates in different moisture levels also showed the same tendency, which indicates the correlation between conidial germination and efficacy of *M. anisopliae*. However, the mortality for MAX-2 decreased much more slowly than those of the other isolates. At the substrate with 8% moisture, which was too low for *M. anisopliae* to facilitate germination, MAX-2 still showed medium mortality of 41% versus low mortality < 5% for the other isolates against *T. molitor* larvae. Howard et al*.*[15] observed that high virulence of *M. anisopliae* against mosquitoes is not significantly affected by low viability, and they deduced that the difference is possibly due to the different abilities of the fungal conidia to germinate on mosquito cuticles and the agar. Leger [16] also reported the existence of two diverse sets of selection pressures on *Metarhizium* spp., one for optimum characteristics for soil survival and another for virulence to insects. Although desiccation stress restricted the viability of MAX-2 on the substrate, this isolate might have more excellent ability to germinate and infect after attachment to the host by its own high trehalase activity, some other mechanism, or the induction of the hosts. Further studies on the possible mechanisms of water stress response and high efficacy for MAX-2 are recommended.

Sporulation of entomopathogenic fungi is significantly affected by moisture content, commonly between 1:0.35 and 1:0.60 (wet substrate: water) in mass production, of the solid substrate [17]. The optimum moisture levels of the substrate for *M. anisopliae* range from 57% to 58% [18]. In the present study, conidial germination and the efficacy of *M. anisopliae* were tested with a dry substrate at moisture levels from 8% to 35%, at which all isolates caused 100% mortality, except for MAQ-28 (95% mortality). The moisture contents of substrates decreased as water evaporated over time. To avoid contamination, the moisture levels were determined by testing the initial moisture contents of the substrates before inoculation. This study was conducted to test the efficacy of *M. anisopliae* under desiccation stress. The substrates become drier over the testing course, and the tested efficacies of the isolates might be slightly negative for the tested moisture levels.

### Infection characteristics of MAX-2 under desiccation stress

*M. anisopliae* invades and infects the body of an insect by direct penetration of the cuticle or breathing apertures, ingestion into the digestive tract, or wounds [19]. The infected insects lose their appetite and exhibit somewhat sluggish behavior. Some changes in color might be observed shortly before death. At high humidity, the hyphae emerge through the cuticle and form a hyphal layer on the surface of the insect, and the conidium then emerges after death [20,21]. The outward signs of infection on *T. molitor* larvae inflicted with *M. anisopliae* isolate MAX-2 under desiccation stress differed from those in the wet microhabitat. The treated larvae showed dark black internodes and fungal growth after death in the wet microhabitat. However, local black patches appeared on the cuticles and the cadavers dried, and no fungal growth after death was observed under desiccation stress. This phenomenon was possibly due to the possible production of defense measures by the larvae against a finite number of conidia, which had contact with the larvae in the dry microhabitat. Insects usually activate polyphenol oxidase and melanize their cuticles when wounded or infected with microbial pathogens to heal wounds or prevent microbial intrusion [22]. The local black patches on *T. molitor* larvae in the dry microhabitat could come from their own polyphenol oxidase activity or resistance to other pathogens. This phenomenon was supported by the few larvae that survived and exuviated, leaving the shell with local black patches (Figure 3i). The wet substrate allowed the production of mass mycelia and conidia, which added to the initial inoculum concentration and increased the penetration efficiency. In addition, the mycelia and its toxin released in the substrate could be ingested by the larvae. The larvae had little chance to protect against invasion, and no local black spots were found. This observation was supported by the high mortality in the wet microhabitat for all isolates. Whether the different symptoms suggest diverse infection mechanisms to *T. molitor* larvae is worthy of further investigation.

### Efficacy of M. anisopliae isolate against pests under desiccation environment

As an alternative to chemical control, the use of fungal insecticides for the biological control of insect pests has attracted significant interest. However, entomopathogenic fungi have not achieved wide-scale use in agriculture in spite of their apparent efficacy in small-scale field trials, mainly because they require high humidity and temperature to grow and disperse. *M. anisopliae* is a common soil-borne entomopathogenic fungus that is found worldwide, and environmental factors affect its persistence and activity. Moisture level is a major factor that affects the ability of fungi to survive, propagate, and infect and kill their host [23]. The field moisture level usually does not satisfy the requirements for germination and growth of *M. anisopliae*[24]*.* Studies on drought tolerance, which is a key part of stress tolerance, are important for the use of fungi in biocontrol [5,25]. Our results indicate that *M. anisopliae* isolate MAX-2 maintained high efficacy under desiccation stress, and exhibited great potential for development.

The isolate was obtained from Shangri-la in Yunnan, China. This region is at high altitude with an extensive annual arid period, high UV radiation, and dry and windy weather. The fungi might have developed desiccation tolerance to adapt to the extreme environment, such as low humidity. The tolerance of this fungus to other stressors needs further investigation. The characteristics of MAX-2 provide genetic resources of resistance, and indicate the potential of developing a biopesticide from the fungal isolate for managing pests under desiccation stress.

## Conclusion

The efficacies of four *M. anisopliae* isolates from arid regions of Yunnan Province in China were tested. A valid laboratory bioassay system was established to study *M. anisopliae* efficacy under desiccation stress with sterile *T. molitor* larvae in substrates with low moisture content. The infective capacity of *M. anisopliae* isolate MAX-2 under desiccation stress was evaluated using this system. The four isolates showed gradient descent efficacies and gradient descent capacities against desiccation. MAX-2 showed significantly higher efficacy and higher antistress capacity than the other isolates under desiccation stress. MAX-2 caused different symptoms on *T. molitor* larvae under desiccation stress and in the wet microhabitat. The larvae showed local black patches on the cuticles, and the cadavers dried without mycelia or conidia under desiccation stress. The internodes of insects became dark, and mycelium and conidia formed on the cadavers in the wet microhabitat. Our findings could encourage further investigation and development of *M. anisopliae* isolate MAX-2, and attract research interest on the stress tolerance of biocontrol fungi.

## Methods

### Solid substrates

Wheat bran substrates with different moisture levels were used in this study. The substrates were sterilized at 121°C for 20 min. Sterile wheat bran without water was used as a dry substrate to test the efficacy of *M. anisopliae* under desiccation stress. The moisture contents of substrates were adjusted by adding a certain amount of water and heating 5 g of the sterilized substrate at 100°C for 4 h. Moisture content was then calculated using the dry and initial weights. Moisture content of the dry substrate was determined to be 8%. The gradient of the substrates from the initial moisture content was adjusted to 15%, 20%, 25%, 30%, and 35%.

### Sterile culture of host insects

*T. molitor* larvae were selected as host insects because they can remain active under desiccation stress, and are easily reared under laboratory conditions. Such conditions are convenient for testing the virulence of fungal pathogens under desiccation stress.

To eliminate the effect of some possible microbes, we cultured the host insects under sterile conditions. *T. molitor* larvae were washed in sterile water, and the water on the surface was absorbed using sterile filter papers. The cuticles of the larvae were wiped carefully with 75% alcohol cotton balls for seconds and transferred to sterile filter paper to dry in air for 5 min. Sterilized larvae were reared, incubated, and subcultured in sterile glass jars containing the wheat bran substrate with 15% moisture content.

### Screening of MAX-2 with the capacity of infecting under desiccation stress

#### M. anisopliae isolates in the experiment

*M. anisopliae* isolates were collected from the arid regions of Yunnan Province in China during the dry season. The efficacy test was conducted in the wet substrate with 30% moisture content at 25°C. The isolates MAC-6, MAL-1, and MAQ-28, whose efficacies showed gradient descent, were chosen as controls to display the efficacy of MAX-2 under desiccation stress. The MAX-2 isolate was from Shangri-la, MAC-6 was from Chuxiong, MAL-1 was from Lanping, and MAQ-28 was from Qujing.

#### Conidial production and inoculation

The conidia of *M. anisopliae* isolates were produced by incubating the fungi on potato dextrose agar plates at 25°C for 14 d. Conidia powder of MAX-2 was obtained from the surface of fungal colonies using a sterile scoop and transferred to a sterile tube (20 mm?×?200 mm). Conidial powder was weighed and mixed with sterile wheat bran substrates. The conidial concentration was adjusted to 5?×?10^8^ conidia/g, and the substrates were cultured at 25°C. The conidial concentration was controlled by adjusting the amount of conidial powder in the substrate, and determined by diluting 1 g of the mixture (conidial powder and substrate) with sterile water. The conidia were then counted microscopically using a blood count board.

#### Germination rate assessment at different moisture levels

The conidial germination rates of *M. anisopliae* isolates were assessed on wheat bran substrates (5?×?10^8^ conidia/g) with different moisture contents of 8%, 15%, 20%, 25%, 30%, and 35% at 24 h. The cultivated mixture was obtained from the top to bottom using a sample collector after 24 h of culture, and serially diluted with sterile water to count the conidia microscopically using a blood count board. A conidium is considered to be germinated when its germ tube is equal to at least half of the long axis of the conidium [26]. The germination rate was calculated based on the summation of germinated and nongerminated conidia. At least 300 conidia were counted in the field of view.

#### Efficacy of M. anisopliae isolates against T. molitor larvae at different moisture levels

The eighth to ninth instar larvae of *T. molitor* with similar sizes were used to test and evaluate the efficacy of different fungal isolates (Figure 1f). The efficacies of *M. anisopliae* isolates were determined at various moisture levels (8%, 15%, 20%, 25%, 30%, and 35%). *T. molitor* larvae were placed in glass jars containing the substrates with different moisture contents, which were inoculated with *M. anisopliae* (5?×?10^8^ conidia/g) and cultured at 25°C. The efficacy assay was based on the hosts’ mortality rate 15 d after inoculation. Five replicates were used for every treatment, with 20 larvae in a glass jar for each treatment. Cultures of *T. molitor* larvae in blank substrates (without *M. anisopliae* applied treatments) with the corresponding moisture contents were prepared as negative controls.

The mortality data of *T. molitor* from the tested isolates at different moisture levels were corrected using Abbott’s formula [27], and transformed to arcsine square root values for ANOVA using SPSS software (SPSS version 17.0). Duncan’s new multiple range test was used to determine and compare the means. Differences were considered statistically significant at *P* < 0.05.

#### Infection characteristics of MAX-2 under desiccation stress

The infection processes of MAX-2 in dry and wet microhabitats were observed and compared. The substrate with low moisture content (8%) was used as the dry microhabitat, whereas the substrate with high moisture content (35%) was used as the wet microhabitat. The photographs of the disease symptoms were recorded using a Fujifilm FinePix S1770 camera.

## Competing interests

XL and CZH invented of a patent, for the sterile cultivation method of mealworms (application no. 201110360999.7). The authors declare no competing interests concerning this work.

## Authors’ contributions

CZH and XL conceived of the study, participated in its design and coordination, performed the experiments, and drafted the manuscript. YFL performed the statistical analysis and participated in the design, coordination, and revision of the manuscript. JGH, YJ, and WJY helped in sampling and data collection. All the authors read and approved the final manuscript.

## References

[B1] Mancebo A Gonzalez F Lugo S Gonzalez B Bada A Aldana L Gonzalez Y Arteaga M Fuentes D Toxicity/pathogenicity of metarhizium anisopliae LMA-06 by means of oral and intranasal dosing Pakistan J of Biological Sciences 2005 8 7 969 973

[B2] Arthurs S Thomas MB Effect of temperature and relative humidity on sporulation of Metarhizium anisopliae var. acridum in mycosed cadavers of Schistocerca gregaria J Invertebr Pathol 2001 78 59 65 10.1006/jipa.2001.5050 11812107

[B3] Benjamin MA Zhioua E Ostfeld RS Laboratory and field evaluation of the entomopathogenic fungus Metarhizium anisopliae (Deuteromycetes) for controlling questing adult Ixodes scapularis (Acari: Ixodidae) J Med Entomol 2002 39 723 728 10.1603/0022-2585-39.5.723 12349854

[B4] Bukhari T Takken W Koenraadt CJ Development of metarhizium anisopliae and beauveria bassiana formulations for control of malaria mosquito larvae Parasit Vectors 2011 4 23 10.1186/1756-3305-4-23 21342492 PMC3051916

[B5] Hallsworth JE Magan N Water and temperature relations of growth of the entomogenous fungi beauveria bassiana, metarhizium anisopliae and paecilomyces farinosus J Invertebr Pathol 1999 74 261 266 10.1006/jipa.1999.4883 10534413

[B6] Damir ME Effect of growing media and water volume on conidial production of beauveria bassiana and metarhizium anisopliae J of Biological Sciences 2006 6 2 269 274 10.3923/jbs.2006.269.274

[B7] McCoy CW Baker RR, Dunn PE Entomopathogenic fungi as microbial pesticides New directions in biological control 1990 New York: Liss 139 159

[B8] Arzumanov T Jenkins N Roussos S Effect of aeration and substrate moisture content on sporulation of *Metarhizium anisopliae* var. *acridum* Process Biochem 2005 40 3–4 1037 1042

[B9] Ihara F Yaginuma K Kobayashi N Mishiro K Sato T Screening of entomopathogenic fungi against the brown-winged green bug, Plautia stali Scott (Hemiptera: Pentatomidae) Appl Entomol Zool 2001 36 4 495 500 10.1303/aez.2001.495

[B10] Luo Z Zhang Y Jin K Ma J Wang X Pei Y Construction of beauveria bassiana T-DNA insertion mutant collections and identification of thermosensitive and osmosensitive mutants Acta Microbiol Sin 2009 49 10 1301 1305 20069875

[B11] Qin W Walker VK Tenebrio molitor antifreeze protein gene identification and regulation Gene 2006 367 142 149 16316726 10.1016/j.gene.2005.10.003

[B12] Clopton RE Janovy J Jr Developmental niche structure in the gregarine assemblage parasitizing tenebrio molitor J Parasitol 1993 79 5 701 709 10.2307/3283608

[B13] Clopton RE Janovy J Jr Percival TJ Host stadium specificity in the gregarine assemblage parasitizing Tenebrio militor J Parasitol 1992 78 2 334 337 10.2307/3283484 1556647

[B14] Daoust RA Ward MG Roberts DW Effect of formulation on the viability of Metarhizium anisopliae conidia J Invertebr Pathol 1983 41 2 151 161 10.1016/0022-2011(83)90214-8 6841995

[B15] Howard AK Koenraadt CJ Farenhorst M Knols BG Takken W Pyrethroid resistance in anopheles gambiae leads to increased susceptibility to the entomopathogenic fungi metarhizium anisopliae and beauveria bassiana Malar J 2010 9 168 10.1186/1475-2875-9-168 20553597 PMC2898789

[B16] St. Leger RJ Vurro M, Gressel J M*etarhizium anisopliae* as a model for studying bioinsecticidal host pathogen interactions Novel biotechnologies for biocontrol agent enhancement and management 2007 New York: Springer 179 204

[B17] Leng Y Peng G Cao Y Xia Y Genetically altering the expression of neutral trehalase gene affects conidiospore thermotolerance of the entomopathogenic fungus Metarhizium acridum BMC Microbiol 2011 11 1471 2180 10.1186/1471-2180-11-32 PMC3045870 21310069

[B18] Damir ME Variation in germination, virulence and conidial production of single spore isolates of entomopathogenic fungi in response to environmental heterogeneity J of Biological Sciences 2006 6 2 305 315 10.3923/jbs.2006.305.315

[B19] Gopal M Gupta A Thomas GV Prospects of using metarhizium anisopliae to check the breeding of insect pest, oryctes rhinoceros L. In coconut leaf vermicomposting sites Bioresour Technol 2006 97 15 1801 1806 10.1016/j.biortech.2005.09.005 16230009

[B20] Wang B Zheng J Huang D Wang D Han X Wang X Symptoms and histopathological study of *Anoplophora glabripennis* larvae infected with *Metarhizium* (Metsch.) Sorokin MS01 Front Agric China 2009 3 2 152 158 10.1007/s11703-009-0024-z

[B21] Kassimatis EJM Evaluation of Metarhizium anisopliae mycoinsecticide as an alternative locust control measure in southern Africa PhD thesis 2010 23 University of Pretoria: Zoology and Entomology Department

[B22] Tseng MN Chung PC Tzean SS Enhancing the stress tolerance and virulence of an entomopathogen by metabolic engineering of dihydroxynaphthalene melanin biosynthesis genes Appl Environ Microbiol 2011 77 13 4508 4519 10.1128/AEM.02033-10 21571888 PMC3127726

[B23] Hussein KA Abdel-Rahman MAA Abdel-Mallek AY Climatic factors interference with the occurrence of beauveria bassiana and metarhizium anisopliae in cultivated soil Afr J of Biotechnol 2010 9 45 7674 7682

[B24] Gillespie AT Grawford E Samson RA, Vlak JM, Peters D Effect of water activity on conidial germination and mycelial growth of *Beauveria bassiana*, *Metarhizium anisopliae*, *Paecilomyces* spp. and *Verticillium lecanii* Fundamental and applied aspects of invertebrate pathology 1986 Wageningen: Society of Invertebrate Pathology 254

[B25] Milner RJ Staples JA Lutton GG The effect of humidity on germination and infection of termites by the Hyphomycete, Metarhizium anisopliae J Invertebr Pathol 1997 69 64 69 10.1006/jipa.1996.4636 9028930

[B26] Moore D Langewald J Obognon F Effects of rehydration on the conidial viability of Metarhizium flavoviride mycopesticide formulations Biocontrol Sci Technol 1997 7 87 94 10.1080/09583159731072

[B27] Abbott WS A method of computing the effectiveness of an insecticide J Econ Entomol 1925 18 265 267

